# Indoor airborne bacterial communities are influenced by ventilation, occupancy, and outdoor air source

**DOI:** 10.1111/ina.12047

**Published:** 2013-05-24

**Authors:** J F Meadow, A E Altrichter, S W Kembel, J Kline, G Mhuireach, M Moriyama, D Northcutt, T K O'Connor, A M Womack, G Z Brown, J L Green, B J M Bohannan

**Affiliations:** 1 Biology and the Built Environment Center, Institute of Ecology and Evolution, University of OregonEugene, OR, USA; 2 Department of Biological Sciences, University of QuebecMontreal, QC, Canada; 3 Energy Studies in Buildings Laboratory, Department of Architecture, University of OregonEugene, OR, USA; 4 Department of Ecology and Evolutionary Biology, University of ArizonaTucson, AZ, USA; 5 Santa Fe InstituteSanta Fe, NM, USA

**Keywords:** Bioaerosol, Airborne bacterial community, Natural ventilation, Built environment, Indoor microbial ecology

## Abstract

Architects and engineers are beginning to consider a new dimension of indoor air: the structure and composition of airborne microbial communities. A first step in this emerging field is to understand the forces that shape the diversity of bioaerosols across space and time within the built environment. In an effort to elucidate the relative influences of three likely drivers of indoor bioaerosol diversity – variation in outdoor bioaerosols, ventilation strategy, and occupancy load – we conducted an intensive temporal study of indoor airborne bacterial communities in a high-traffic university building with a hybrid HVAC (mechanically and naturally ventilated) system. Indoor air communities closely tracked outdoor air communities, but human-associated bacterial genera were more than twice as abundant in indoor air compared with outdoor air. Ventilation had a demonstrated effect on indoor airborne bacterial community composition; changes in outdoor air communities were detected inside following a time lag associated with differing ventilation strategies relevant to modern building design. Our results indicate that both occupancy patterns and ventilation strategies are important for understanding airborne microbial community dynamics in the built environment.

## Practical Implications

Building design can have a substantial effect on occupant health and energy use through ventilation strategy, yet we are just beginning to understand the potential microbial community dynamics affected by building design. In this study, we investigated the interrelationships between occupancy, ventilation strategy, and airborne microbial community composition through time in university classrooms. Commercial spaces are often operated so as to be unventilated overnight and on weekends (unoccupied times) in the name of energy efficiency, and this study is the first to identify the remnant microbial signature of under-ventilated classrooms overnight. Passive overnight ventilation, mimicked by the night-flushed treatment in this study, is an energy-efficient way to simultaneously cool building mass and avoid overnight and weekend microbial community stagnancy.

## Introduction

Microorganisms are ubiquitous in the built environment; not only do they cover virtually all surfaces we contact, they also cover our skin and are abundant in the air we breathe. More than 10^6^ bacterial cells per m^3^ are present in both outdoor and indoor air (Kembel et al., 2012), but our understanding of the factors that drive the composition of these assemblages in the built environment is nascent (Hospodsky et al., 2012; Womack et al., 2010).

Sources of airborne bacteria in the built environment are not well known but include humans, pets, soils, and plants, both as direct sources and indirectly from dust perturbations (Bowers et al., 2011a,2011b, 2012; Täubel et al., 2009; Womack et al., 2010). Bacterial communities in outdoor air are highly ephemeral and can change over short periods of time with climatic conditions (Fierer et al., 2008; Rintala et al., 2008; Womack et al., 2010), but the influence of outdoor air variability on indoor air bacterial communities is unknown. Previous research has indicated that human occupancy increases the airborne bacterial load and leaves a distinctly human microbial signal inside of buildings (Hospodsky et al., 2012; Qian et al., 2012), and natural ventilation does influence airborne bacterial community composition in the absence of active human occupants (Kembel et al., 2012), but we currently have little understanding of the relative influences of ventilation and human occupancy on bioaerosols in modern buildings. Nor do we have an understanding of the degree of connectivity between indoor and outdoor airborne microbial communities.

Architectural ventilation design choices have long been recognized to influence human health and productivity (Fisk, 2000; Mendell and Smith, 1990; Seppänen and Fisk, 2002; Sternberg, 2009; Wargocki et al., 2000). In the United States, health and productivity losses attributable to commercial ventilation systems have been estimated in the tens of billions of dollars (Fisk, 2000). Building ventilation in the United States was estimated to consume approximately 1.75 exajoule (4.1% of total US buildings' energy use) in 2010, and that number increases by an order of magnitude when heating and cooling are included (U.S. Department of Energy, 2011). Our improved understanding of the energy implications of ventilation design choices has impacted modern architectural design (Brown and DeKay, 2001; Li et al., 2007; Sternberg, 2009). Previous microbiological research into the effects of indoor ventilation choices, however, has largely focused on known allergens and disease-related microorganisms, but little is known about how ventilation design influences microorganisms in a community context.

Whole microbial communities have historically been problematic to study due to their immense diversity and to a propensity in the field of microbiology to focus on easily culturable microbial species. This has changed considerably in recent years given rapid advances in high-throughput DNA sequencing technologies that allow the generation of relatively comprehensive microbial community data from environmental samples (Caporaso et al., 2012; Hamady et al., 2008). One of the implications of a community-based perspective is the increased understanding that the vast majority of microorganisms on and around humans are not pathogens, but are rather commensals and even mutualistic. While these methods have been widely applied to relatively high-biomass microbial habitats like soil and aquatic environments, application in indoor air research has been slower, partly owing to ultra-low microbial biomass available for DNA assay from airborne indoor samples. Several groups (e.g., Hospodsky et al., 2012; Kembel et al., 2012) have effectively applied 454 pyrosequencing to study indoor air samples using various collection methods, but temporal resolution has consistently been a limiting factor in indoor bioaerosol community surveys.

We conducted an extensive study over a 9 day period in a University of Oregon classroom building to assess the relative influences of natural ventilation and human occupancy on airborne bacterial communities. Our sampling regime was designed for relatively fine temporal resolution that allowed us to observe microbial community changes throughout each day. We employed barcoded Illumina amplicon sequencing of 16S ribosomal RNA genes to capture a comprehensive look at airborne bacterial communities. Specifically, we aimed to answer three questions: (i) What is the degree of connectivity between indoor and outdoor airborne bacterial communities? (ii) are differing choices in ventilation strategy associated with detectable temporal changes in indoor airborne bacterial community composition? and (iii) is classroom occupant load predictive of indoor airborne bacterial community composition?

## Methods

### Sample collection

All air samples were collected at the Lillis Business Complex, University of Oregon, Eugene, OR, USA, from August 1–10, 2011. Eight classrooms were surveyed throughout the study period (mean floor area = 121.1 m^2^ ± 14.4 s.d.; mean room volume = 468.94 m^3^ ± 55.71 s.d.). Each air sample consisted of two 25 mm-diameter cellulose ester filters (1.4 μm pore diameter; autoclaved prior to sample collection) housed in paired SKC Button Samplers (SKC Inc., Eighty Four, PA, USA). Air filters each passed approximately 4 l per min for 8 h, for a total air volume of approximately 1.92 m^3^. Air was drawn using an AirChex XR5000 pump (also manufactured by SKC Inc.) housed in a custom noise-reducing box to facilitate sample collection during classroom occupancy. At each 8-h time period, 12 pairs of filter samples were running simultaneously: four in separate classrooms on the first floor (subsequently referred to as ‘night-flushed’); four in separate classrooms on the second floor (‘non night-flushed’); and four at separate outdoor corners of the Lillis Business Complex (‘outside’). Indoor filters were positioned on desktops (approximately 90 cm above the floor); outdoor filters were atop pump housing boxes (approximately 30 cm above ground level). All rooms received a combination of unfiltered outdoor air and mechanically ventilated, filtered air from the building's HVAC system. All mechanically ventilated air (supplied by the building's HVAC system) passed through MERV-8 filtration. Night-flushed rooms are distinguished by receiving maintained unfiltered outdoor ventilation air, while ventilation in non-night-flushed rooms depended on hybrid, programed building algorithms similar to standard commercial building operation. The ventilation and occupancy treatments are depicted in detail in Figure S1. All classrooms had sheet linoleum flooring; none were carpeted. Technicians were on-site throughout the sampling period monitoring classroom occupancy, and air filter samples were frozen at -80°C immediately following sampling and stored frozen until processing. Ventilation rates were collected for each room from the building's direct digital control monitoring system. Wind data were compiled from the National Weather Service's weather station at Mahlon Sweet Field (EUG; 13 km NW of the Lillis Business Complex).

### DNA amplification and sequencing

Whole genomic DNA was extracted using the MO BIO PowerWater DNA Isolation Kit (MO BIO Laboratories, Carlsbad, CA, USA) according to manufacturer's instructions with the following modifications: air filters were incubated with Solution PW1 in a 65°C water bath for 15 min prior to bead beating; bead beating length was extended to 10 min; and samples were eluted in 50 μl Solution PW6. Paired filters underwent DNA extraction together.

The V4 region of the 16S rRNA gene was amplified using F515/R806 primer combination (5′-GTGCCAGCMGCCGCGG-3′, 5′-TACNVGGGTATCTAATCC-3′) (Caporaso et al., 2012; Claesson et al., 2010). Amplification proceeded in two steps using a custom Illumina preparation protocol described in Meadow et al. (2013), where PCR1 was performed with forward primers that contained partial unique barcodes and partial Illumina adapters. The remaining ends of the Illumina adapters were attached during PCR2, and barcodes were recombined *in silico* using paired-end reads. Adapter sequences are detailed in supplemental materials. All extracted samples from paired air filters were amplified in triplicate for PCR1, and triplicates were pooled before PCR2. PCR1 (25 μl total volume per reaction) consisted of the following steps: 5 μl 5X HF buffer (Thermo Fisher Scientific, Waltham, MA, USA), 0.5 μl dNTPs (10 mm; Invitrogen, Grand Island, NY, USA), 0.25 μl Phusion Hotstart II polymerase (0.5 units; Thermo Fisher Scientific.), 13.25 μl certified nucleic acid–free water, 0.5 μl (10 μm) forward primer, 0.5 μl (10 μm) reverse primer, and 5 μl template DNA. The PCR1 conditions were as follows: initial denaturation for 2 min at 98°C; 22 cycles of 20 s at 98°C, 30 s at 50°C and 20 s at 72°C; and 72°C for 2 min for final extension. After PCR1, the triplicate reactions were pooled and cleaned with the QIAGEN MinElute PCR Purification Kit according to the manufacturer's protocol (QIAGEN, Germantown, MD,USA). Samples were eluted in 11.5 μl of Buffer EB (10 mm Tris-Cl, pH 8.5, Qiagen). For PCR2, a single primer pair was used to add the remaining Illumina adaptor segments to the ends of the concentrated amplicons of PCR1. The PCR2 (25 μl volume per reaction) consisted of the same combination of reagents that was used in PCR1, along with 5 μl concentrated PCR1 product as template. The PCR2 conditions were as follows: 2 min denaturation at 98°C; 12 cycles of 20 s at 98°C, 30 s at 66°C and 20 s at 72°C; and 2 min at 72°C for final extension.

Amplicons were size-selected by gel electrophoresis: gel bands of target length were extracted and concentrated, using the ZR-96 Zymoclean Gel DNA Recovery Kit (ZYMO Research, Irvine, CA, USA), following manufacturer's instructions. DNA concentrations were quantitated using a Qubit Fluoromoeter (Invitrogen), and samples were pooled in equimolar concentrations for library preparation prior to sequencing. Samples were sent to the Dana-Farber/Harvard Cancer Center DNA Resource Core (Boston, MA, USA; dnaseq.med.harvard.edu) and sequenced on the Illumina MiSeq platform as paired-end reads.

### Sequence processing

Raw sequences were processed using the FastX Toolkit (http://hannonlab.cshl.edu/fastx_toolkit) and the QIIME pipeline (Caporaso et al., 2010). Barcodes were recombined from paired-end reads, and the reverse reads were used for downstream analysis. All sequences were trimmed to 140 bp, including a 12-bp barcode, and low-quality sequences were removed. Quality filtering settings were as follows: minimum 30 quality score over at least 75% of the sequence read; no ambiguous bases allowed; 1 primer mismatch allowed. After quality control and barcode assignment, the remaining high-quality sequences were binned into operational taxonomic units (OTUs) at a 97% sequence similarity cutoff using UCLUST (Edgar, 2010). The highest-quality sequences from each OTU cluster were taxonomically identified using BLAST against reference sequences from Greengenes (DeSantis et al., 2006). Plant chloroplast and mitochondrial OTUs were removed. Not all samples returned the same number of sequences, so we rarefied all samples to 900 sequences per sample. Samples with fewer than 900 sequences were not used in subsequent analyses. Sequence files and metadata for all samples used in this study have been deposited in the public FigShare data repository (http://dx.doi.org/10.6084/m9.figshare.157199).

### Statistical analysis

All statistical analyses were performed in R (R Development Core Team, 2010), primarily utilizing the vegan, labdsv, and picante packages (Kembel et al., 2010; Oksanen et al., 2011; Roberts, 2010). Phylogenetic diversity (Faith's PD) was calculated using the pd function in picante. Community variation among samples, or β-diversity, was calculated using the quantitative, taxonomy-based Bray-Curtis dissimilarity, implemented in the vegan package. To focus on the effect of night ventilation, we analyzed a subset of the data coinciding with a shift in outdoor microbial community composition; the 25 most abundant OTUs were used for a trimmed dataset from eight consecutive time periods (Thursday 12 a.m.–Saturday 4 p.m.). OTU relative abundances were displayed using spline curves from actual relative abundances as a way to more clearly visualize community changes. These 25 abundant taxa were also identified by hand using BLASTn to attain more confident classifications. Distance-based redundancy analysis (DB-RDA) was used to test for an effect of occupancy on community composition and was performed with the capscale function in vegan. Three relatively abundant human-associated bacterial genera (*Corynebacterium, Staphylococcus,* and *Acinetobacter*) were selected to detect a human signal (Grice and Segre, 2011; Hospodsky et al., 2012; Qian et al., 2012). These three genera encompass taxa that occur in diverse habitats, but all three also contain taxa that have been previously associated with human skin, and thus were used as a surrogate for human presence. These were selected from OTUs based on taxonomic assignments from the Greengenes reference database.

## Results

**Fig 1:**
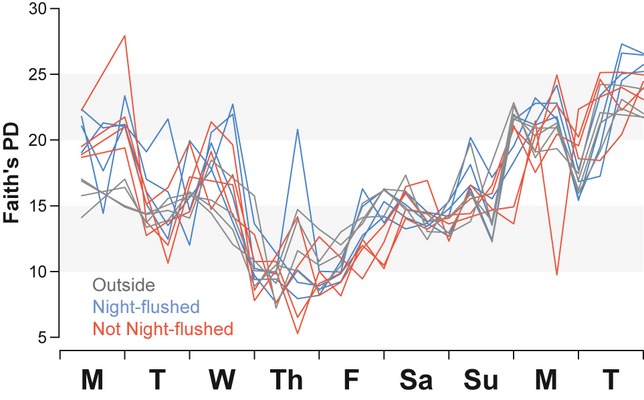
Indoor air phylogenetic diversity closely resembles outdoor air. Faith's phylogenetic diversity (PD) values for all samples (900 sequences per sample) are overlaid throughout the week. Outdoor air experienced temporal community changes throughout the sampling period, and this outdoor influence is experienced inside of rooms regardless of ventilation strategy

After quality filtering, 4 756 624 sequences were clustered into 10 782 OTUs, of which 275 400 sequences were randomly selected to represent 306 air samples, at a 900 sequence per sample rarefaction level. After rarefaction, approximately half (50.8%) of all OTUs were represented by only one or two sequences (so-called singletons and doubletons). The two most common OTUs detected were *Sphingomonas* spp. (4.1% & 3.1% of rarefied sequences). Two *Acinetobacter*-related OTUs were among the 25 most common detected inside classrooms (2.3% of all indoor sequences), but these were less common outside (<1% of outdoor sequences). Overall, we found substantial overlap between indoor and outdoor communities; 88 of the 100 most common indoor OTUs were also among the 100 most common outdoors. Additionally, outdoor phylogenetic diversity was closely mirrored indoors throughout the sampling period (Figure 1).

### Occupied versus unoccupied classrooms

**Fig 2:**
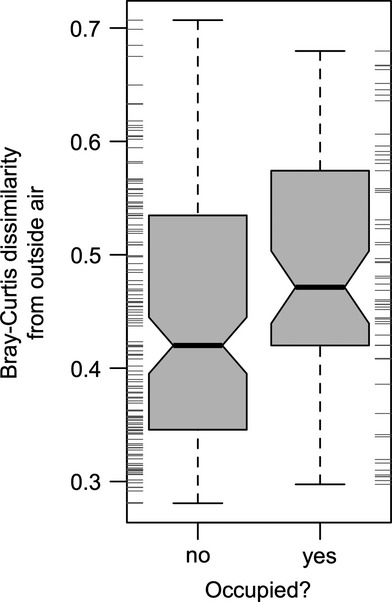
Bacterial communities in unoccupied rooms were more similar to concurrent outdoor air than occupied rooms. When all indoor samples are compared with outdoor air samples taken during the same time period, occupied rooms were more dissimilar (*P* = 0.018). Marginal tick marks show the distribution of dissimilarity values in either group. Box plots delineate (from bottom) minimum value, Q1, median (Q2), Q3, and maximum value; notches approximate 95% confidence around median values

Classrooms in the Lillis Business Complex were generally occupied during the 8 a.m.–4 p.m. sampling period from Monday–Thursday (mean maximum occupant load = 28.8; see Figure S1); occupancy was lower on Friday (mean = 10.7), and no classrooms were occupied over the weekend. Airborne bacterial communities in unoccupied rooms were significantly more similar to concurrent outdoor air than were occupied rooms (*P* = 0.018; *t* = 2.38; from a two-sample Welch's *t*-test on Bray-Curtis Dissimilarities; Figure 2). Room occupancy was a significant predictor of community composition over the 9-day sampling period (*P* = 0.005; *F* = 2.37; from DB-RDA MANOVA on Bray-Curtis Dissimilarities), although occupancy only explained a small amount of the total variance (1.2%). When we focused on three major potentially human-associated bacterial OTU genera, *Corynebacterium, Staphylococcus*, and *Acinetobacter*, all three were significantly more relatively abundant inside than outside (Table 1). Two of the three were also more abundant in occupied classrooms than in unoccupied classrooms.

**Table 1:** Human-associated bacterial genera were more common inside and in occupied rooms

Comparison	OTU Genus	df^a^	*T*-value	*P*-value
In^3^ vs. out	*Corynebacterium*	272.98	7.6	<0.001
	*Staphylococcus*	276.4	2.61	0.009
	*Acinetobacter*	303.53	4.2	<0.001
Occupied vs. unoccupied	*Corynebacterium*	76.88	1.95	0.054
	*Staphylococcus*	173.27	2.09	0.038
	*Acinetobacter*	203.21	4.48	<0.001

Tests are based on log-transformed relative abundances to normalize abundance distributions.

a Degrees of freedom were estimated due to unequal variance.

b All indoor samples, regardless of occupation, are included.

### Ventilation strategy

**Fig 3:**
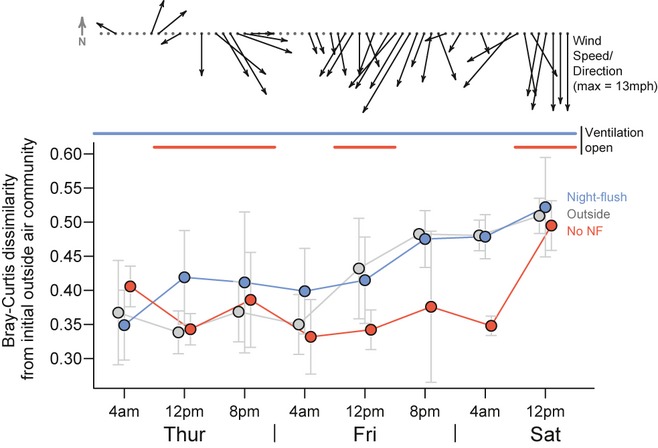
Unventilated rooms retain legacy of outdoor air communities. Gray points on the bottom panel show the average outdoor community dissimilarity (±1 s.d.) from each initial outdoor sample at the beginning of the sampling period shown (Thursday 12 a.m.–8 a.m.); blue and red points represent fully ventilated (Night-flush) and conventionally ventilated (No Night-flush) classrooms, respectively. Blue and red bars above the points show ventilation regime, with night-flush (blue) vents open throughout the sampling period, and non-night-flushed (red) vents only open during occupied hours. Arrows show hourly wind direction and speed (length) during the sampling period

Ventilation management in commercial buildings (including university buildings) is complex and often based on a combination of expected occupancy and indoor/outdoor temperatures. The Lillis Business Complex integrates both filtered mechanically ventilated air, and unfiltered outdoor air introduced through dampers in exterior walls. We manipulated four of the sampled classrooms to operate as a ‘night-flushed’ treatment (see Figure S1) with direct outdoor ventilation inlets fully open (average 5.87 room changes per hour ± 2.28 s.d., when dampers were open). The other four classrooms (‘non night-flushed’) were ventilated with hybrid, programed building algorithms that are similar to standard commercial building operation. Outdoor ventilation inlets in these rooms were open during the 8 a.m.–4 p.m. sampling period and closed from 12 a.m.–8 a.m., and generally open for only the beginning of the 4 p.m.–12 a.m. sampling period. We manipulated outdoor ventilation inlets on Friday evening to effect closed ‘non-nightflushed’ classrooms for 16 consecutive hours; this coincided with a detectable shift in outdoor airborne bacterial communities. These classrooms experienced essentially no unfiltered outdoor air for the duration of the 16 h, although filtered mechanical ventilation remained active until 7:00 p.m. Figure 3 shows air samples during a 3-day period expressed by dissimilarity from initial outdoor air communities (*t*_0_ = Thursday 12 a.m.–8 a.m.; self comparisons were excluded to avoid zero values). A community shift in outdoor air was observed during the day on Friday, and the same community shift can be seen almost simultaneously in the night-flushed classrooms. The ventilation dampers in the non-night-flushed rooms, however, were closed off during the night and open during occupied hours, a standard practice in commercial building operation. Because the shift in outdoor communities occurred when the non-ventilated rooms were closed to outside air, the community shift is not fully detected in these classrooms for another 16 h, when the ventilation dampers are opened again. Although changes in outdoor airborne microbial communities are poorly understood, the shift seen in outdoor air in Figure 3 coincides with an increase in sustained wind speed.

**Fig 4:**
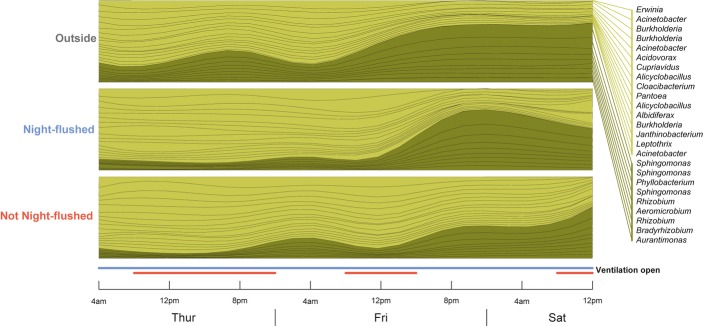
Twenty-five most abundant OTUs exhibit the temporal community changes detected in Figure 3. Relative abundances of the 25 most common bacterial taxa (mean = 42.6% of sequences ± 8.5 s.d.) shift at different rates based on ventilation regime. The height of each taxon band is equivalent to the relative abundances of each taxon, and they are colored by whether they were more abundant during the beginning of the sampling period or at the end. OTU names are from BLAST identification to the genus level, and all identifications shown were ≥99% similar to reference NCBI sequences. Red bars indicate the status of ventilation dampers in non-night-flushed rooms; dampers in night-flushed rooms were open throughout the sampling period shown

The time lag associated with this difference in ventilation strategy can be seen manifested in the most abundant bacterial OTUs; Figure 4 shows the community shift as expressed by the 25 most common OTUs based on their mean relative abundances in the three air sources. The apparent community change that occurs in outdoor air samples happens shortly after in night-flushed classrooms and not completely in the non-night-flushed classrooms for another 16 h.

## Discussion

This study illustrates the highly interrelated nature of two major drivers of indoor airborne microbial community composition: human occupancy and ventilation strategy. Both have previously been implicated individually (Hospodsky et al., 2012; Kembel et al., 2012), but they have not been studied together. Our results show that, while occupancy does increase a human signal in the detectable airborne community, indoor air tends to closely mirror outdoor air in a well-ventilated space regardless of occupancy.

Human occupancy in buildings resuspends settled dust, and humans also shed substantial bacterial biomass during normal activity (Hospodsky et al., 2012; Qian et al., 2012), but the present study indicates that this human-associated contribution to the total detectable airborne microbial community is perhaps less important than ventilation strategy and air source. Occupancy in this study was a significant but relatively weak predictor of airborne community composition and was most obvious when considering only human-associated genera. This was somewhat diminished by the fact that the three focal human-associated genera in this study never accounted for more than 38% (mean = 7.8%) of observed sequences in occupied rooms.

Ventilation source alters microbial communities, regardless of ventilation rates, in a hospital setting (Kembel et al., 2012), but mechanical ventilation in a hospital employs more stringent air filtration than in typical commercial buildings, including university buildings. The building in the present study was designed to utilize unfiltered natural ventilation supplemented by mechanically filtered supply air; this use of unfiltered air was apparent in the close resemblance between outdoor and indoor airborne communities. Phylogenetic diversity of indoor and outdoor air was indistinguishable throughout the sampling period and followed a remarkably similar pattern. Although comparable phylogenetic diversities certainly do not equate to identical communities, 88% of the most common indoor species were also the most common outdoor species, and these tended to fluctuate together. Given this strong coupling between indoor and outdoor airborne microbial communities, it is also likely that seasonal variation in outdoor bioaerosols (Bowers et al., 2012) as well as indoor dust (Rintala et al., 2008) would be reflected in bioaerosols indoors. Localized seasonal weather patterns, however, dictate ventilation strategies, and our results indicate that ventilation strategy influences indoor/outdoor bioaerosol connectivity.

Ventilated indoor spaces have long been recognized as more beneficial to human health and productivity than relatively stagnant spaces, and this has been variously attributed to comfort, humidity, reduced accumulation of indoor aerosol pollution, and reduced contact with human-associated disease-causing agents (Fisk, 2000; Mendell and Smith, 1990; Seppänen and Fisk, 2002; Sternberg, 2009; Wargocki et al., 2000). Recent evidence even points to a link between exposure to outdoor biodiversity and reduced early development of asthma and allergies (Ege et al., 2011; Hanski et al., 2012; Heederik and Von Mutius, 2012; Perzanowski et al., 2012). Modern design principles often incorporate passive natural ventilation as a means of effecting energy-efficient air turnover, and our results suggest a microbial community corollary from this approach.

## Conclusions

Ventilation strategy and human occupancy in a university building were shown to influence indoor airborne bacterial communities. In this hybrid-ventilated building, indoor air closely mirrored outdoor air in terms of bacterial community composition over time. When rooms were unventilated, vestige outdoor airborne microbial communities were detectable even after communities in adjacent, ventilated rooms changed along with outdoor air. Well-ventilated spaces are desirable from a health and productivity standpoint, and while human occupancy does result in a human-associated airborne microbial fingerprint, room ventilation can affect microbial community turnover so as to more closely resemble outdoor air.

## Supporting Information

Fig S1 Occupancy data (a) and damper position (b) are displayed for all time periods and rooms considered in this study.
