# Supplementary material for: Indoor airborne bacterial communities are influenced by ventilation, occupancy, and outdoor air source
Source: Indoor Air. 2013 May 24;24(1):41–8. doi: 10.1111/ina.12047 (PMC4285785; doi:10.1111/ina.12047)
Supplement: Fig S1 — Occupancy data (a) and damper position (b) are displayed for all time periods and rooms considered in this study. [file ina0024-0041-SD1.doc]

SUPPLEMENTAL MATERIALS - Indoor Air

Indoor Airborne Bacterial Communities Are Influenced By Ventilation, Occupancy, and Outdoor Air Source.

*Meadow JF* , Altrichter AE, Kembel SW, Kline J, Mhuireach G, Moriyama M, Northcutt D, OConnor TK, Womack AM, Brown GZ, Green JL, Bohannan BJM*

*Corresponding author: [jfmeadow@gmail.com](mailto:jfmeadow@gmail.com)


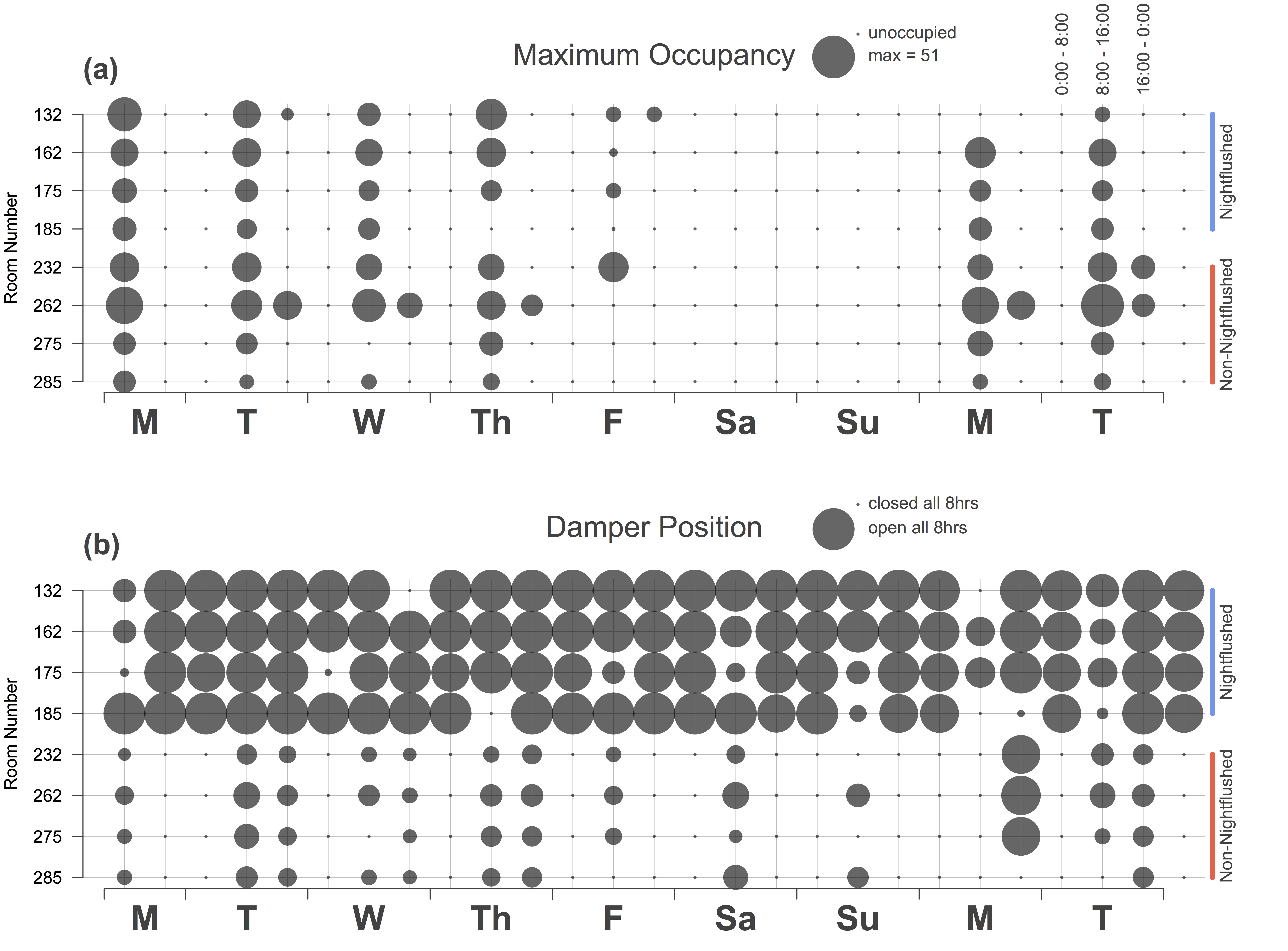


Supplemental Figure 1. Occupancy data (a) and damper position (b) are displayed for all time periods and rooms considered in this study. Bubble size indicates relative value for either variable

**Primer/Adapter Sequences:** Illumina adapters were included only partially during the first PCR, and extended during a second PCR using the sequences below.

Adapter + primer sequences for PCR1:

5’ TCTCGGCATTCCTGCTGAACCGCTCTTCGATCT-XXXXXX- GTGCCAGCMGCCGCGG 3’ 5’ ACACTCTTTCCCTACACGACGCTCTTCCGATCT-XXXXXX- TACNVGGGTATCTAATCC 3’

*where XXXXXX = 6bp barcode added to each primer*

Adapter addition sequences for PCR2:

5’ AAGCAGAAGACGGCATACGAGATCGGTCTGGCATTCCTGC 3’

5’ ATGATACGGCGACCACCGAGATCTACACTCTTTCCCTACACGACG 3’
